# Total Replacement of Soybean Meal with Sundried Soymilk Residue in the Total Mixed Ration has a Negative Impact on Intake, Digestibility, and Milk Production in Dairy Goats

**DOI:** 10.1155/2024/7441866

**Published:** 2024-03-02

**Authors:** Thaintip Kraiprom, Sitthisak Jantarat, Suphawadee Yaemkong, Narakamol Laorodphan, Nithat Wichasit, Muhammad Khan, John Mauck, Juan J. Loor, Tossaporn Incharoen

**Affiliations:** ^1^Faculty of Science and Technology, Prince of Songkla University, Pattani 94000, Thailand; ^2^Faculty of Food and Agricultural Technology, Pibulsongkram Rajabhat University, Phitsanulok 65000, Thailand; ^3^Faculty of Agriculture Natural Resource and Environment, Naresuan University, Phitsanulok 65000, Thailand; ^4^Department of Animal Nutrition, University of Veterinary and Animal Science, Lahore, Pakistan; ^5^Department of Animal Sciences, University of Illinois, Urbana 61801, USA

## Abstract

This study aimed to evaluate whether total replacement of soybean meal (SBM) with sundried soymilk residue (SSR) in a total mixed ration (TMR) affects intake, digestibility, milk production, and blood metabolites in dairy goats. A total of 12 healthy Saanen dairy goats (40.12 ± 5.80 kg of BW) in midlactation (31.23 ± 10.12 days) were used in a randomized complete design (*n* = 4 goats/group). Dietary treatments were based on a TMR as follows: control TMR without SSR (CON) or SBM-based TMR with 50% or 100% of SSR replacing SBM (SSR-50 and SSR-100, respectively). All goats had ad libitum access to feed and clean water throughout the experiment. The dry matter (DM) intake decreased (*p* < 0.05) with the increasing replacement ratio of SBM and was lowest in the SSR-100 group. Similarly, organic matter (OM) digestibility was lowest (*p* < 0.05) in the SSR-100 group. However, the digestibility of DM, CP, NDF, and ADF did not change (*p* > 0.05) by dietary treatments. Compared with CON, the milk yield decreased significantly (*p* < 0.05) with increasing replacement ratio of SBM. In contrast, milk composition such as total solids, solids-not-fat, milk fat, lactose, protein, and pH were not influenced (*p* > 0.05) by feeding dietary SSR. Compared with other treatments, blood glucose concentration was lower (*p* < 0.05) in the SSR-100 group. In contrast, packed cell volume, glucose, and plasma urea nitrogen concentrations did not differ (*p* > 0.05). The results indicated that SSR could replace SBM in a TMR at less than 50%. Thus, the present study provides support for further investigation to enhance the utilization of soybean waste as an alternative protein source in the TMR for dairy goats and potentially other ruminants.

## 1. Introduction

Over the past decade in Thailand, goats have been considered an important ruminant species, primarily attributed to their demonstrated economic impacts as proficient converters of low-quality feed into meat and milk. Thus, dairy goat farming offers a promising livestock option for numerous small-scale agricultural enterprises and rural households. Park et al. [1] reported that goat milk generates finer and softer curds compared with cow milk when subjected to acidification conditions similar to those within the stomach, thus enhancing its digestibility. Goat milk also serves as an essential dietary choice for individuals who suffer from cow milk allergies. Because the proteins found in goat milk are less abundant in *α*s1-casein, the protein causes the majority of allergic responses to cow's milk [2]. This seems that the market opportunity for goat milk has led to its growing popularity. Currently, it is possible to make a wide range of product from goat milk. These include dairy beverage products, fermented products (cheese, buttermilk, or yogurt), ice cream, sweets and candies, and other specialist items such as hair care, skin care, and cosmetics [3]. However, feeding costs are the most important expense in dairy goat production systems. As such, discovering sources of alternative feedstuffs derived from locally agroindustrial wastes could be important for enhancing the profitability of dairy goat farming.

In dairy production, balancing the goal of maximizing milk production while minimizing protein inputs has become a critically important aspect in recent years. Because protein is a relatively expensive nutrient, excessive dietary protein can result in unnecessary feeding expenses, ultimately reducing the overall profitability of dairy producers [4]. Furthermore, excessive protein intake can lead to reduced efficiency of nitrogen use for milk protein and increased nitrogen excretion in manure [5, 6]. Soybean meal (SBM) is the most commonly used protein supplement in dairy rations due to its high digestible protein source, amino acid profile, and palatability. However, in the post-COVID-19 period, the increase in price of protein sources, and particularly SBM, have imposed challenges for nutritionists attempting to design least-cost diet formulations. Thus, searching for alternative protein sources is an ongoing focus.

Sundried soymilk residue (SSR) or okara is a byproduct of soymilk and tofu manufacturing, which contains the insoluble material remaining after the filtration of soy slurry from the aqueous extract. It is considered a viable substitute for a portion of protein in ruminant rations. Fresh SSR has been used as a feed ingredient to substitute SBM without a negative impact on feed intake and productivity in various ruminants including goats [7, 8], beef steers [9], and lactating ewes [10]. In terms of dry matter (DM), SSR contains a significant amount of metabolizable energy (ME; 9.0–14.2 MJ/kg) and various components such as crude protein (209.0–391.0 g/kg DM), neutral detergent fiber (241.0–726.0 g/kg DM), ether extract (49.0–215.0 g/kg DM), and ash (34.0–53.0 g/kg DM) [11–13]. Thus, from a nutritional standpoint, replacing SBM with SSR in dairy goat rations could result in decreased feeding costs without harming productivity.

Currently, modern dairy producers recognize that total mixed rations (TMRs) represent an effective strategy in terms of optimal use of nutrients by ruminants. These dietary formulations are principally characterized by the harmonized integration of roughage and concentrates, thereby ensuring precision in nutrient delivery and yielding optimal production performance [14]. Our hypothesis was that SSR may completely replace SBM without having a detrimental impact on whole productivity of dairy goats. The specific objectives were to determine the effects of total replacement of SBM with SSR in the TMR on intake, digestibility, milk production, and blood metabolites in dairy goats.

## 2. Materials and Methods

### 2.1. Ethical Statement

The experimental procedures were conducted in accordance with the Ethical Principles and Guidelines for the Use of Animals for Scientific Purposes, National Research Council of Thailand. All animal care and management implemented in this study were approved by the Institutional Animal Care and Use Committee at Prince of Songkla University (ref. 40/2018), Thailand.

### 2.2. Animal, Diets, and Experimental Design

A total of 12 vaccinated and healthy Saanen dairy goats (40.12 ± 5.80 kg of BW) at midlactation (31.23 ± 10.12 days) were divided into 3 groups with 4 replications. The goats were assigned to the treatments based on live body weight and daily milk yield at the outset of the experiment. They were housed in individual stalls with free access to feed and clean water daily. A mineral block was available for ad libitum intake. The dietary treatments were based on TMR as follows: control TMR without SSR (CON) or the SBM-based TMR replaced with 50% or 100% of SSR (SSR-50 and SSR-100, respectively) (Table 1). The TMR comprised of 50% Napier grass silage with a particle size ranging from 1 to 2 cm and combined with 50% concentrate (on DM basis). The concentrate contained conventional ingredients (ground corn, rice bran, molasses, urea, dicalcium phosphate, and salt) and different levels of SSR. Crude protein (CP), organic matter (OM), ether extract (EE), ash, and DM were analyzed on these feed samples according to AOAC [15]. Neutral detergent fiber (NDF) and acid detergent fiber (ADF) were determined based on the method suggested by Van Soest [16].

### 2.3. Milk Collection and Analysis

Each goat was hand milked two times daily (07.00 A.M. and 05.00 P.M.) from 14 days postexperiment and reported as a one-day milk sample throughout the 90-day experimental period. Before milking, the nipple was washed and disinfected with 70% alcohol to prevent microbial contamination. Collected milk was carefully weighed using a digital balance and recorded to calculate the yields. For milk composition assessment, morning milk samples were collected weekly from 14 to 63 days postexperiment. Milk pH was immediately measured after milking. Milk samples (approximately, 150 mL) were conserved with bronopol (2-bromo-2-nitropropane-1,3-diol) and transported to the laboratory using a cooler at 4°C. These samples were analyzed for fat, protein, lactose, total solids, fat, and solids-not-fat using infrared spectroscopy (Milko Scan 133, Foss Electric, Denmark).

### 2.4. Feed Intake and Digestibility Measurements

Throughout the feeding trial, feed offered and refused for individual animals were recorded daily before the morning feeding. Each goat was fed twice daily (06.00 A.M. and 04.00 P.M.) before milking. Feed intake was determined by subtracting the amount of feed offered to each goat from the feed refused within a 24-hour period. In the final week of the experiment, rectal fecal samples were collected daily into plastic bags from each animal for 5 consecutive days before the morning feeding. Feed (residual and supplied) was sampled daily and placed in labeled plastic containers. These samples were stored frozen at −20°C until the end of the sample collection period. The feces from each individual goat were consolidated to form a single sample. The collected feed obtained from each goat was pooled together and treated as 1 sample. Both samples (feces and feed) were dried in a hot-ventilated oven at 60°C for 48 h to reach a constant weight. Before chemical analysis, dried samples were finely ground to pass through a 1-mm screen in a Cyclotec laboratory mill (Tecator, Hoganas, Sweden) and stored in sealed plastic bags kept in a humidity-controlled box. All samples were subjected to analysis for DM, CP, and OM, following the procedures outlined by AOAC [15]. In addition, the concentrations of NDF and ADF were determined in accordance with the methodology described by Van Soest [16]. The parameters of digestibility (DM, CP, OM, NDF, and ADF) were calculated using the following equation: apparent digestibility was determined as ((Amount ingested − Amount excreted)/(Amount ingested)) × 100.

### 2.5. Blood Metabolites

On the last day of the digestibility trial period, blood samples were collected from the jugular vein of each goat at both 0 and 4 hours after feeding, and each vial separated into two sets (approximately, 5 mL) according to method described by Kraiprom et al. [17]. The blood samples were placed in a vial coated with ethylene diamine tetraacetic acid (EDTA) after which a microhaematocrit capillary tube was used to determine packed cell volume (PCV) using a Hemocrit reader. The second set of blood samples was collected in vials without EDTA for assessment of glucose concentration and plasma urea nitrogen (PUN). Then, the samples were subjected to centrifugation at 1500 × *g* for 10 min to isolate plasma and the tube was subsequently kept frozen at −20°C until further analysis. The glucose concentration was assessed through an enzymatic colorimetry assay kit (Sigma Aldrich, St. Louis, MO, USA). The concentration of PUN was assessed according to the urea liquicolor test (HUMAN GmbH, Wiesbaden, Germany) via spectrophotometry at a wavelength of 578 nm.

### 2.6. Statistical Analysis

The experiment was conducted as a completely randomized design (CRD). All collected data were subjected to statistical analysis using one-way analysis of variance (one-way ANOVA) performed with the Statistical Package for the Social Sciences (SPSS), version 17.0 (SPSS Inc., Chicago, USA). The results are reported as the means for each treatment, accompanied by the pooled standard error of the means (SEMs). Differences among treatments were evaluated using Duncan's multiple range test and significance was determined at a probability level of *p* < 0.05.

## 3. Results

### 3.1. Feed Intake and Digestibility

Feed intake and apparent nutrient digestibility are reported in Table 2. The DM intake decreased (*p* < 0.05) with increasing replacement ratio of SBM and was lowest in the SSR-100 group. Similarly, OM digestibility was lower (*p* < 0.05) in the SSR-100 group compared with other treatments. However, the digestibility of DM, CP, NDF, and ADF did not change (*p* > 0.05) due to treatments.

### 3.2. Milk Production and Components

Milk production-associated parameters are reported in Table 3. Compared with the CON group, the milk yield of dairy goats decreased significantly with the increasing replacement ratio of SBM (*p* < 0.05). However, milk total solids, solids-not-fat, fat, lactose, protein, pH, and density were not affected (*p* > 0.05) by feeding dietary SSR.

### 3.3. Blood Metabolites

Characteristics of blood metabolites are reported in Table 4. Although blood glucose levels before feeding did not differ among treatments, the SSR-100 group had lower (*p* < 0.05) concentrations compared with other groups. However, there were on significant differences in blood glucose levels at 4 hr postfeeding among the groups. Across all treatments, the average levels of glucose, PCV, and PUN were comparable (*p* > 0.05).

## 4. Discussion

The lower DM intake when SSR was fed, especially SSR-100, was similar to the experiment of Rahman et al. [7] working with male crossbred goats fed with soy waste. The partial replacement of SBM with yeast-derived microbial protein or a TMR with yeast-derived microbial protein also decreased DM intake of lactating cows [18, 19]. The reduction in DM intake with SSR might be attributed to low palatability and sensory factors. Due to these constraints on feed intake, it appeared that SSR could be used to partially replace SBM in a TMR as long as the level of supplementation does not exceed the totality. Support for this idea are the data from Rahman et al. [8] in which no negative impact on feed intake was detected in goats fed soya waste up to 2.0% (DM basis) of body weight over a period of 14 months. Replacement of SBM with 15% soymilk byproduct did not affect DM intake in cows during early to midlactation [20]. Zang et al. [21] investigated replacing SBM with dried soybean curd residue in a TMR and also did not observe negative effects on DM intake of dairy cows. Thus, the data from dairy cows support the idea that a partial replacement of SBM by SSR in dairy goat diets is feasible. Although SSR did not affect the digestibility of CP, NDF, and ADF, the lower OM digestibility in the SSR-100 group might be caused by the low nutritional value and high fiber content of SSR. This supplement contained a significant amount of NDF (425.8 g/kg DM). Support for this idea comes from the study of Na et al. [22] where OM digestibility in goats and deer decreased linearly (*p* < 0.01) with the increasing dietary NDF content.

The lower milk yield in both SSR groups confirmed the biological link between milk output and adequate DM intake and OM digestibility. Work from Durman et al. [23] in which up to 95 g/kg of soybean byproduct could be added to the TMR of lactating cows without affecting the milk yield underscores the idea that there is an upper limit for the inclusion of SSR in dairy goat diets. Metabolically, the lower blood glucose level especially in the SSR-100-fed group likely reflects the decreased availability of nutrients for intestinal absorption due to the lower DM intake. The fact that blood glucose is directly connected with the milk yield, as it was observed that a high glucogenic diet increased daily milk yield in dairy cows (∼5%) [24], is further evidence that at high levels of supplementation, SSR can reduce nutrient supply to the mammary gland of dairy goats and impair production.

## 5. Conclusions

Replacing SBM with SSR at a higher level in the TMR resulted in a detrimental impact on production performance of dairy goats. Therefore, the present data suggest that SSR could substitute SBM in a TMR at less than 50%. Further investigations to determine the optimal level of soybean waste as an alternative protein source in the TMR for dairy goats and other ruminants should be performed.

## Figures and Tables

**Table 1:** Feed ingredients and chemical components of the total mixed ration replacing soybean meal with sundried soymilk residue.

Items	Treatments^1^		
	CON	SSR-50	SSR-100
Feed mixture ratios (%)			
Napier grass silage	50.00	50.00	50.00
Ground corn	13.00	11.00	7.00
Rice bran	12.00	14.00	18.00
SBM	20.00	10.00	0.00
SSR	0.00	10.00	20.00
Molasses	3.25	3.25	3.25
Urea	0.75	0.75	0.75
Dicalcium phosphate	0.50	0.50	0.50
Salt	0.50	0.50	0.50
Chemical composition			
DM, % of fresh matter	60.9	57.5	53.3
OM, g/kg DM	921.1	915.5	906.6
CP, g/kg DM	150.7	147.2	139.7
EE, g/kg DM	31.2	32.3	34.2
NDF, g/kg DM	360.8	395.7	425.8
ADF, g/kg DM	262.6	282.1	302.3
Ash, g/kg DM	78.9	84.5	93.4

^1^The dietary treatments were based on the total mixed ration (TMR) with varying protein contents as follows: CON = control TMR without soymilk residue, SSR-50 = TMR with 50% of the SBM was replaced by soymilk residue, and SSR-100 = TMR with 100% of the SBM was replaced by soymilk residue. SBM = soybean meal; SSR = sundried soymilk residue; DM = dry matter; OM = organic matter; CP = crude protein; EE = ether extract; NDF = neutral detergent fiber; ADF = acid detergent fiber.

**Table 2:** Effect of replacing soybean meal with sundried soymilk residue in the total mixed ration on feed intake and apparent nutrient digestibility in dairy goats.

Items	Treatments^1^			SEM	*p* value
	CON	SSR-50	SSR-100		
Total DM intake (kg/d)	1.94^a^	1.49^b^	0.95^c^	2.10	0.01
DM intake, %BW	4.13^a^	3.94^b^	3.12^c^	0.46	0.04
DM intake, g/kg BW^0.75^	116.01^a^	94.98^b^	69.23^c^	0.51	0.01
Apparent nutrient digestibility (%)					
DM	77.56	76.45	75.34	3.41	0.51
CP	78.61	74.29	73.24	3.42	0.18
OM	77.11^a^	75.72^a^	72.23^b^	0.65	0.03
NDF	79.41	77.32	70.51	2.13	0.12
ADF	72.52	71.23	69.00	32.45	0.65

^1^The dietary treatments were based on the total mixed ration (TMR) with varying protein contents as follows: CON = control TMR without sundried soymilk residue, SSR-50 = TMR with 50% of the SBM was replaced by sundried soymilk residue, and SSR-100 = TMR with 100% of the SBM was replaced by sundried soymilk residue. SEM = standard error of the mean; DM = dry matter; BW = body weight; BW^0.75^ = metabolic body weight; CP = crude protein; OM = organic matter; NDF = neutral detergent fiber; ADF = acid detergent fiber. ^a–c^Means in the same row with different letters differ (*p* < 0.05).

**Table 3:** Effect of replacing soybean meal with sundried soymilk residue in the total mixed ration on the milk yield and composition in dairy goats.

Items	Treatments^1^			SEM	*p* value
	CON	SSR-50	SSR-100		
Milk yield (kg/d)	1.89^a^	1.23^b^	1.12^b^	1.61	0.04
Total solids (%)	13.85	12.81	12.79	1.65	2.18
Solid not fat (%)	8.53	8.41	8.17	1.31	1.60
Fat (%)	5.34	4.43	4.63	2.54	1.59
Lactose (%)	2.98	3.06	2.85	3.21	2.12
Protein (%)	4.53	4.55	4.20	2.45	1.80
pH	6.61	6.65	6.54	2.15	3.13
Density	1.03	1.02	1.02	1.05	2.84

^1^The dietary treatments were based on the total mixed ration (TMR) with varying protein contents as follows: CON = control TMR without sundried soymilk residue, SSR-50 = TMR with 50% of the SBM was replaced by sundried soymilk residue, and SSR-100 = TMR with 100% of the SBM was replaced by sundried soymilk residue. SEM = standard error of the mean. ^ab^Means in the same row with different letters differ (*p* < 0.05).

**Table 4:** Effect of replacing soybean meal with sundried soymilk residue in the total mixed ration on blood metabolized characteristics in dairy goats.

Items	Treatments^1^			SEM	*p* value
	CON	SSR-50	SSR-100		
Glucose (mg/dl)					
0 hr feeding	68.00^a^	71.00^a^	62.00^b^	0.21	0.02
4 hr feeding	71.08	73.49	67.19	1.05	0.16
Mean	69.52	72.22	64.59	2.07	0.31
PUN (mg/dl)					
0 hr feeding	21.35	20.51	18.03	1.20	1.35
4 hr feeding	23.50	21.92	18.45	2.40	1.70
Mean	22.41	21.20	18.24	2.31	1.48
PVC (%)					
0 hr feeding	30.41	31.28	32.00	1.55	0.90
4 hr feeding	29.80	30.21	30.00	1.70	1.87
Mean	30.11	30.74	31.00	0.90	1.39

^1^The dietary treatments were based on the total mixed ration (TMR) with varying protein contents as follows: CON = control TMR without sundried soymilk residue, SSR-50 = TMR with 50% of the SBM was replaced by sundried soymilk residue, and SSR-100 = TMR with 100% of the SBM was replaced by sundried soymilk residue. SEM = standard error of the mean; PUN = plasma urea nitrogen; PCV = packed cell volume. ^ab^Means in the same row with different letters differ (*p* < 0.05).

## Data Availability

The data used to support the findings of this study are included within the article.
